# Photodermatitis and ocular changes in nine horses after ingestion of wild parsnip (pastinaca sativa)

**DOI:** 10.1186/s12917-022-03162-2

**Published:** 2022-02-26

**Authors:** Judith C. Winter, Katharina Thieme, J. Corinna Eule, Eva-Maria Saliu, Olivia Kershaw, Heidrun Gehlen

**Affiliations:** 1 Equine Clinic: Surgery and Radiology, Freie Universität Berlin, Berlin, Germany; 2 Small Animal Clinic, Freie Universität Berlin, Berlin, Germany; 3 Institute of Animal Nutrition, Freie Universität Berlin, Berlin, Germany; 4 Institute of Pathology, Freie Universität Berlin, Berlin, Germany

**Keywords:** Photosensitization, Parsnip, Furocoumarins, Intoxication, Ocular changes, Cornea, Edema, Erythema, Sunburn, Bergapten

## Abstract

**Background:**

Primary photosensitization rarely occurs in horses and can easily be misinterpreted. Descriptions of the disease in horses after ingestion of parsnip are lacking. The aim of this case series was to describe the dermatological and ocular changes due to photosensitization and to raise awareness of parsnip being a possible aetiologic agent.

**Case presentation:**

Nine horses from three different stables in Berlin and Brandenburg, Germany, presented variable degrees of erythema, scaling, crusting and necrosis of unpigmented skin at the head and prepuce. Horses were of different breeds with a median age of 15 ± 5.9 years. A mild leukocytosis was diagnosed in 1/9 horses at admission. Analyzed liver enzymes were within the reference ranges in all horses. Ocular changes were diagnosed as follows: blepharitis (3/9), conjunctivitis (7/9), corneal edema without additional signs of keratitis and/or uveitis (2/9), corneal edema with signs of uveitis (1/9) and photophobia (4/9). One horse developed a fluorescein positive corneal erosion. Skin biopsy (1/9) revealed a moderate to severe acute, eosinophilic and lymphocytic dermatitis with dermal edema and vasculitis. All stables housing these patients fed hay from the same distributer. Analyzed hay samples showed high contents of wild parsnip (plants, seeds, roots). Wild parsnip is widespread in Europe and contains furocoumarins, a family of photodynamic pigments, which may cause primary photodermatitis, keratoconjunctivitis and uveitis. Horses were treated according to severity of clinical symptoms systemically with flunixine meglumine (1.1 mg/kg BW 1-2x/day) or prednisolone (1 mg/kg BW 1x/day). Topically, either gentamicin (3x/day), dexamethasone (2-3x/day) and/or atropine (1x/day) were used. Skin care was provided with almond oil or dexpanthenol (2x/day). All horses were kept in a dark environment or were treated with sunscreen and facemasks. Duration of treatment varied from 6–30 days (median 11.3 days).

**Conclusion:**

Ingestion of wild parsnip (*Pastinaca sativa*) can induce primary photosensitization with dermatitis and ocular injury in horses. In times of extreme weather, hay may alter in botanical composition, resulting in high amounts of uncharacteristic plants causing novel problems.

## Background

Primary (type I) photosensitization is a clinical condition occurring in animals and humans with accumulation of phototoxic compounds in the skin, cornea and/or mucoid membranes after ingestion of or direct dermal contact with plants or chemicals [1, 2]. Photosensitization outbreaks have been reported from 20 different countries in the peer reviewed literature with the highest number of reports from Australia and Brazil and only 12 reports from Europe [3]. Sheep were the most frequently reported species followed by cattle and horses. Of the reported cases, 68.5% were suspected or confirmed hepatogenous (type II) photosensitization and only 22.5% of the cases were diagnosed or suspected to be of the primary type. Different plants have been identified as causal agents, depending on geographic location. St. John’s wort (*Hypericum perforatum)* is common to the United States, South America, Europe, New Zealand, and Australia, *Brachiaria decumbens* has been identified in Brazil, Colombia and Nigeria. Panicum grasses, *Lantana* spp. and the fungus *Pithomyces chartarum* were the most commonly reported etiological agents in Australia [3].

Wild parsnip (*Pastinaca sativa*) belongs to the *Umbelliferae* or *Apiaceae* family that includes celery, parsley and hogweed among others and is widespread in Europe. It prefers direct sunlight and due to the taproot, wild parsnip is draught resistant. Its seeds, flowers and stem contain furocoumarins [4, 5]. Thirteen different furocoumarins were identified in seeds of wild parsnip. The main compound in mature seeds was bergapten (40.8% in fresh weight, synonym 5-Metoxypsoralen, 5-MOP). All other identified furocoumarins in wild parsnip are summarized in Table 1 [6]. Since the 1940s, it is known that the plant can cause photodermatitis in humans and was listed as a primary photosensitizing plant with maximum weight of evidence for definite phototoxicity [7–9]. However, reports on photodermatitis in horses and livestock are scarce [10, 11]. Furocoumarins are the phototoxic agents in wild parsnip. They are a family of photodynamic pigments produced by a variety of plants. When exposed to UV-A radiation (320–400 nm) from sunlight, furocoumarins interact with oxygen and produce ROS (reactive oxygen species). These ROS can damage cell membranes which may lead to edema formation, blistering and epidermal and dermal cell death [4]. To our best knowledge, this case report is the first peer reviewed publication to describe photodermatitis and ocular changes in horses after ingestion of wild parsnip.

**Table 1:** Furocoumarins identified in immature seeds of wild parsnip (Pastinaca sativa). Information from Kviesis et al., 2019

Furocoumarin	Percentage in fresh weight reported in wild parsnip seeds (%)
Bergapten	40.8
Byakangelicol	14.4
Pimpinellin	10.5
Heraclenin	8.5
Phellopterin	7.2
Methoxsalen	5.7
Isopimpinellin	4.3
Imperatorin	3.2
Isobergapten	2.5
Byakangelicin	1.3
Heraclenol	0.5
Psoralen	0.3
Isobyangelicin	0.8

## Case presentation

### Stable 1

**Fig. 1:**
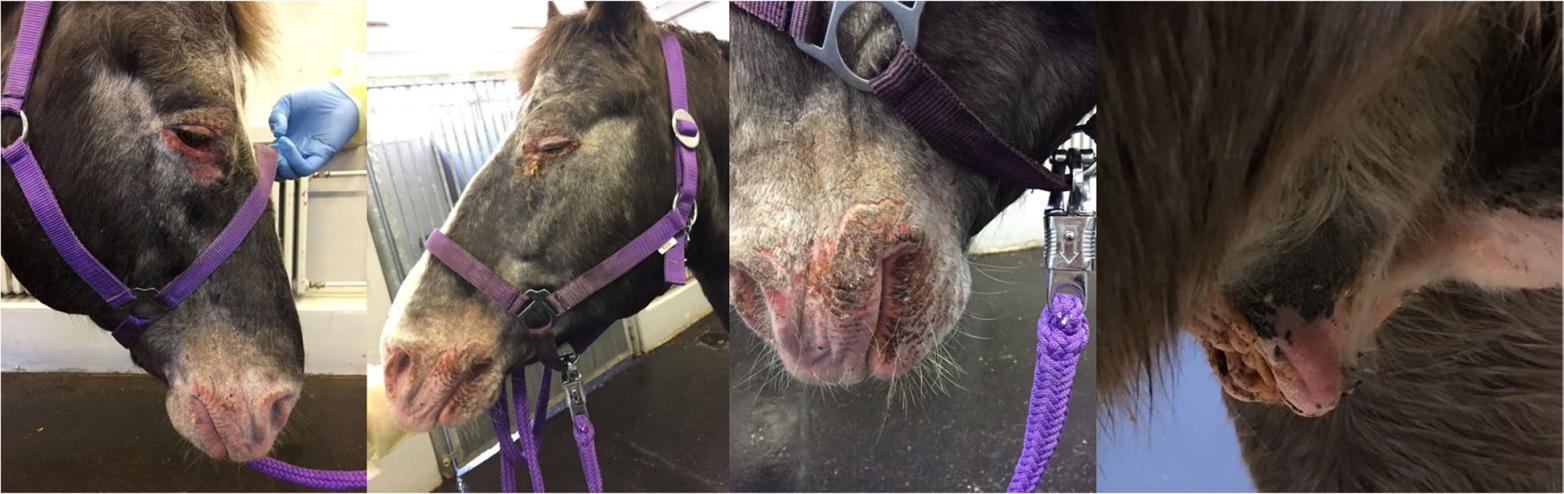
Horse 1 at admission (day 0) showing erythema, crusting and blistering of the unpigmented skin at the head and prepuce

**Fig. 2:**
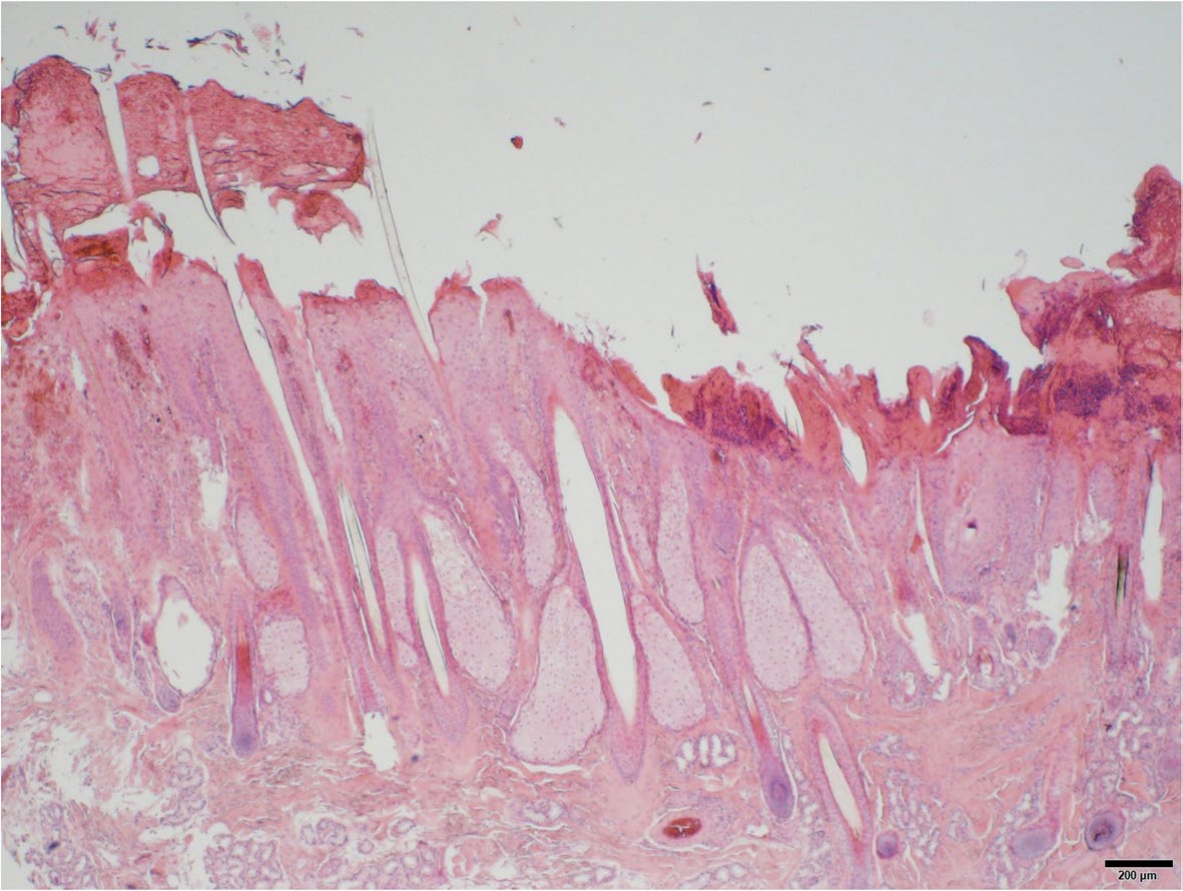
First skin biopsy of horse 1 with detection of acanthocytes. Hematoxylin and eosin (HE)

**Fig. 3:**
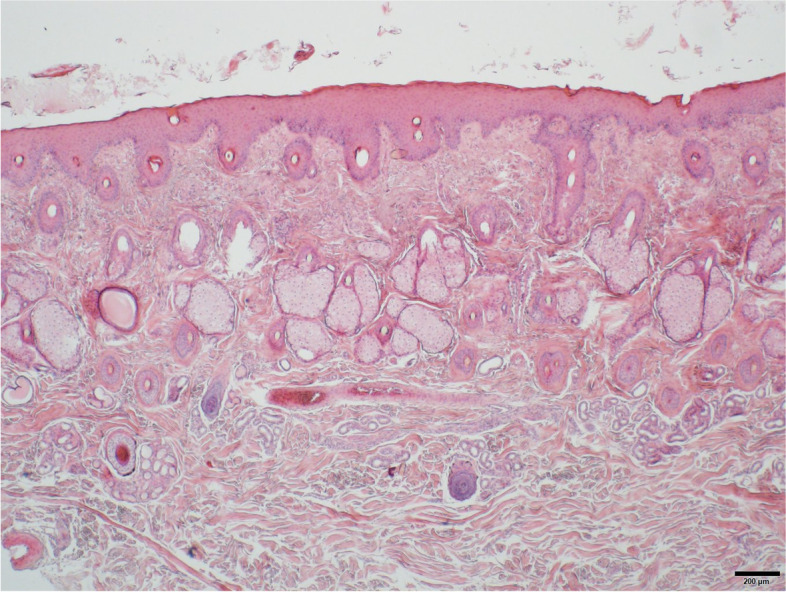
Second skin biopsy of horse 1. The lesion pattern and the lack of acanthocytes led to the suspected diagnosis of hypersensitivity reaction type I. HE

**Fig. 4:**
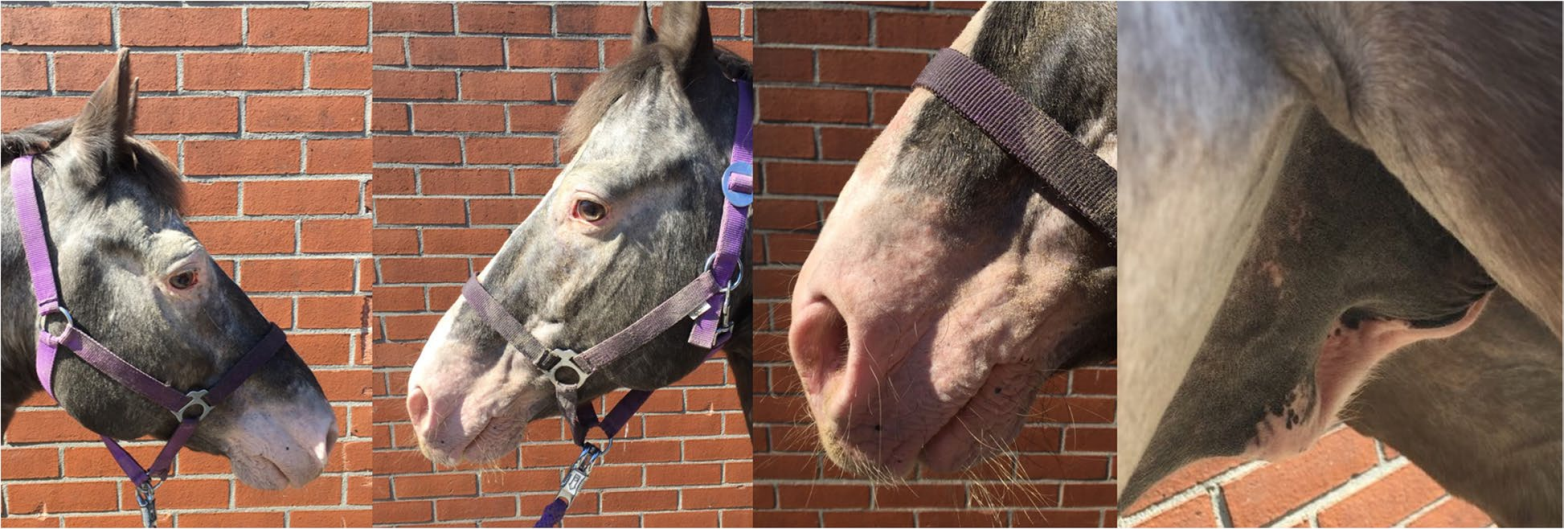
Horse 1 at day 28 without clinical signs of phytophotodermatitis

The first patient (horse 1) was presented to the Equine Clinic at Freie Universität Berlin in February 2019 with blepharospasm and epiphora non-responding to treatment. It was reported that the horse had experienced episodes of the same clinical symptoms since November 2018. The horse was a 23-year-old Appaloosa-mixed breed stallion with large areas of unpigmented skin at the head and prepuce. Treatment by the referring veterinarian included flunixine meglumine (1.1 mg/kg BW, 1x/day, orally) and ofloxacin (2x/day, topically). At admission, the horse showed moderate depression, an increased heart (56 beats/min), and respiratory rate (26 breaths/min). The unpigmented skin at the head and prepuce showed erythema, thickening, scaling and crusting, especially around the eyes and muzzles (Fig. 1). The horse was reluctant to be touched at the head and showed photophobia. No pathological findings besides severe muco-purulent ocular discharge, blepharitis and conjunctivitis were detected within the ophthalmological examination, which included slit-lamp biomicroscopy (SL-14®, Kowa Company, Sakai, Osaka, Japan), rebound tonometry (TonoVet®, icare, Tiolat, Finland), direct ophthalmoscopy (Beta 200®, Heine, Ettenheim, Germany), and fluorescein staining (I-DEW FLO®, ERC Ltd., London, UK). Blood leukocytes (measured in house^a^), GGT, GLDH, AST, Bile acids and ACTH (measured at an external laboratory^b^) were within the reference ranges. The serum selenium- and zinc-concentrations were below the reference ranges (measured at an external laboratory^b^, selenium: 31.6 µg/L (100–200 µg/L); zinc 6.9 µmol/L (9.2–19.9 µmol/L)). Microbiological examination of the skin revealed growth of *Streptococcus dysgalactiae* subsp. *equisimilis*. Skin biopsy showed severe purulent neutrophilic to eosinophilic erosive to ulcerative dermatitis with adhesion of serocellular crusts, epidermal and adnexal hyperplasia and copious amounts of acanthocytes (Fig. 2). A botanical analysis of a hay sample revealed thistle and ribwort besides different grasses. The hay sample was of fair quality with moderate dust concentration. Based on the biopsy results, the suspicion of pemphigus foliaceus was stated. The horse was treated with prednisolone^1^ (1 mg/kg BW, 1x/day, orally) and gentamicin eye ointment^2^ (3x/day, topically). Skin care was provided with a polyhexanid-solution^3^ and almond oil. The horse was supplemented with selenium^4^ according to manufacturer´s instructions. The horse improved significantly throughout the next days and was discharged without clinical symptoms of dermatitis on day 25 after admission. After discharge from the clinic, skin care was provided once daily with almond oil and prednisolone was given in subsequently reduced dosage. Seven days after discharge from the clinic, the horse was presented again with similar symptoms. It was reported that other horses from the same stable showed similar symptoms in unpigmented areas of the skin. Microbiological examination of the skin was repeated and again revealed growth of *Streptococcus dysgalactiae* subsp. *equisimilis* while a new skin biopsy showed a moderate to severe acute, eosinophilic to lymphocytic dermatitis with severe superficial dermal edema, vasculitis and multifocal lysis of collagen fibers (Fig. 3). Acanthocytes were not detected. The suspicion of a hypersensitivity reaction type I was stated, as reported in photodermatitis, while the previously suspected diagnosis of pemphigus foliaceus was not confirmed. A new hay sample could not be examined. The horse was treated symptomatically with prednisolone^1^ (1 mg/kg BW, 1x/day, orally) as before in combination with azathioprine^5^ (0.5 mg/kg BW, 1x/day, orally) and skin care was provided with almond oil. Again, the horse improved rapidly throughout the next days (Fig. 4). The dosage of prednisolone was gradually reduced and ended after 28 days of treatment while azathioprine was continued until day 38. The horse was discharged at day 68 to a new stable and did not show any symptoms afterwards. While treating this patient, two other horses from a different stable (stable 2) were presented to the clinic with similar symptoms.

### Stable 2

**Fig. 5:**
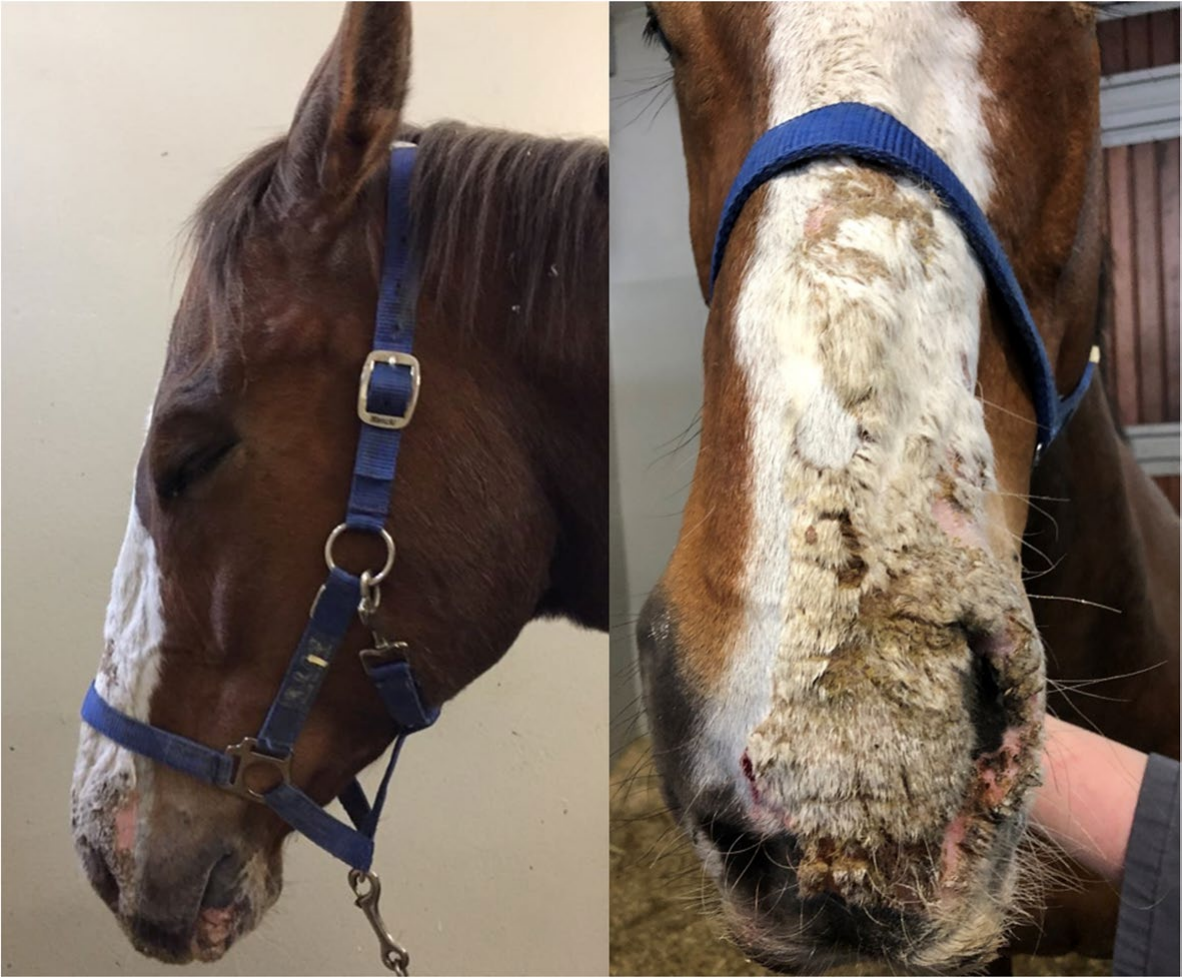
Horse 3 showing photophobia, depression, and dermatitis at admission (day 0)

**Fig. 6:**
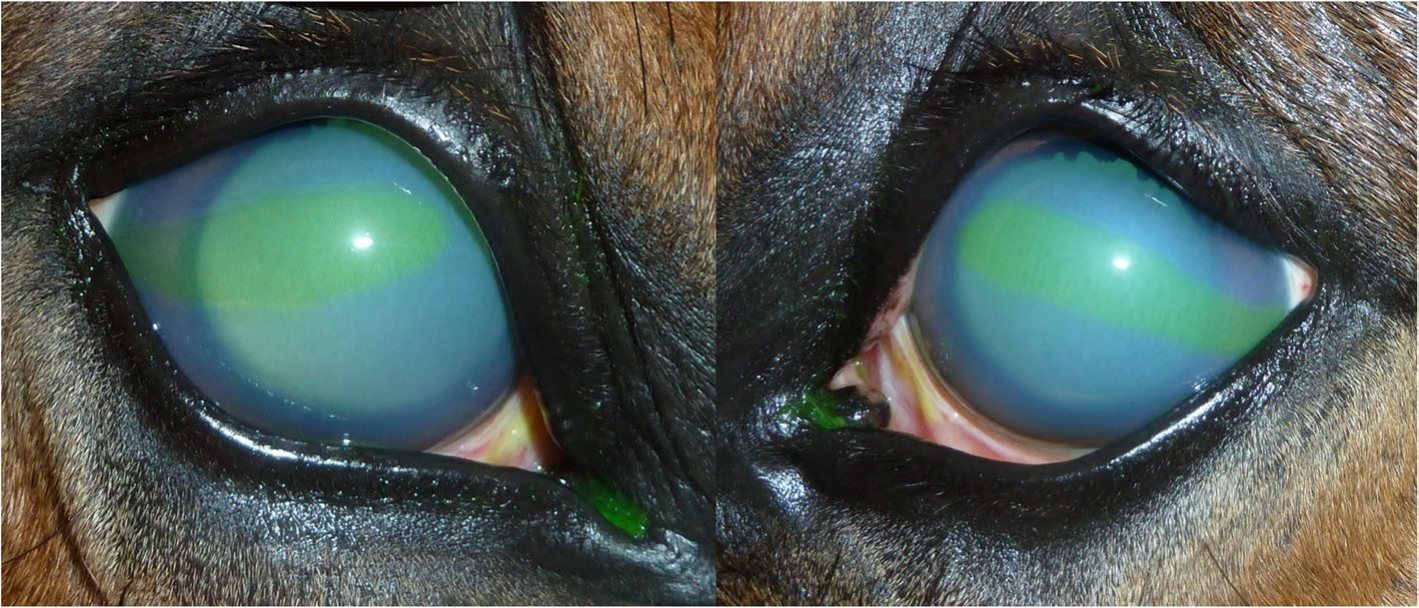
Horse 2 with marked corneal edema and corneal lesions on day 3. Mydriasis is due to the treatment with atropine eyedrops

**Fig. 7:**
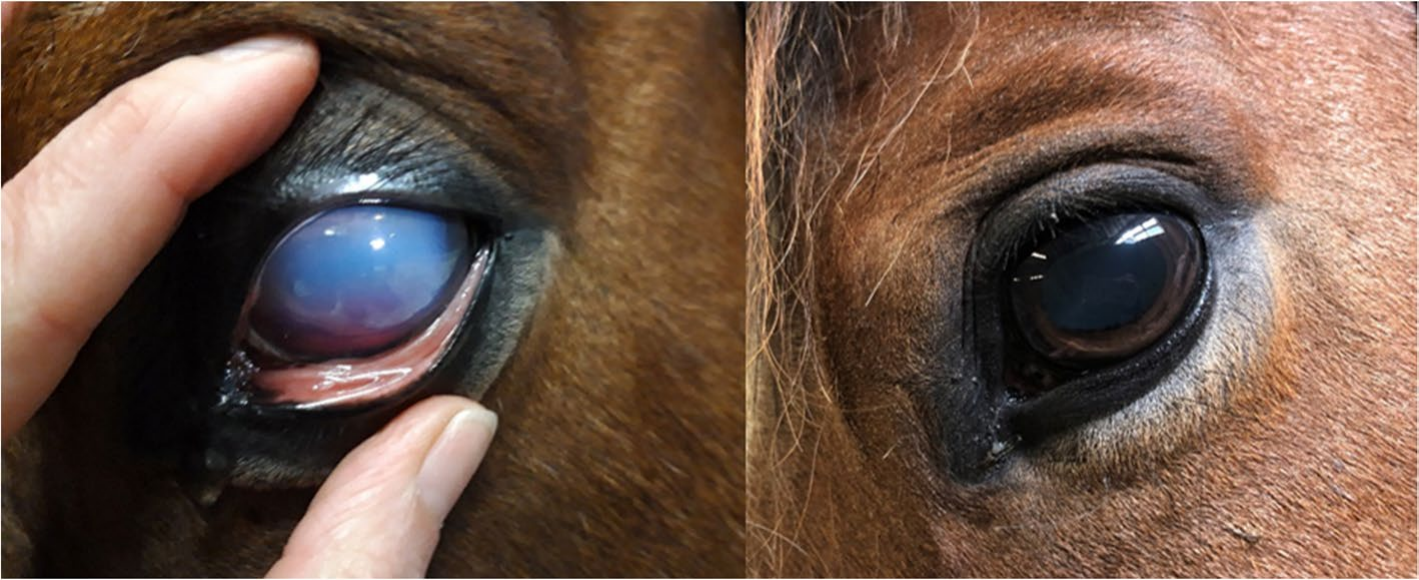
Horse 3 showing moderate corneal edema at admission (day 0) and mild edema at day 24. There is slight red discoloration in the ventral aspect of the cornea, resulting from hyphema

**Fig. 8:**
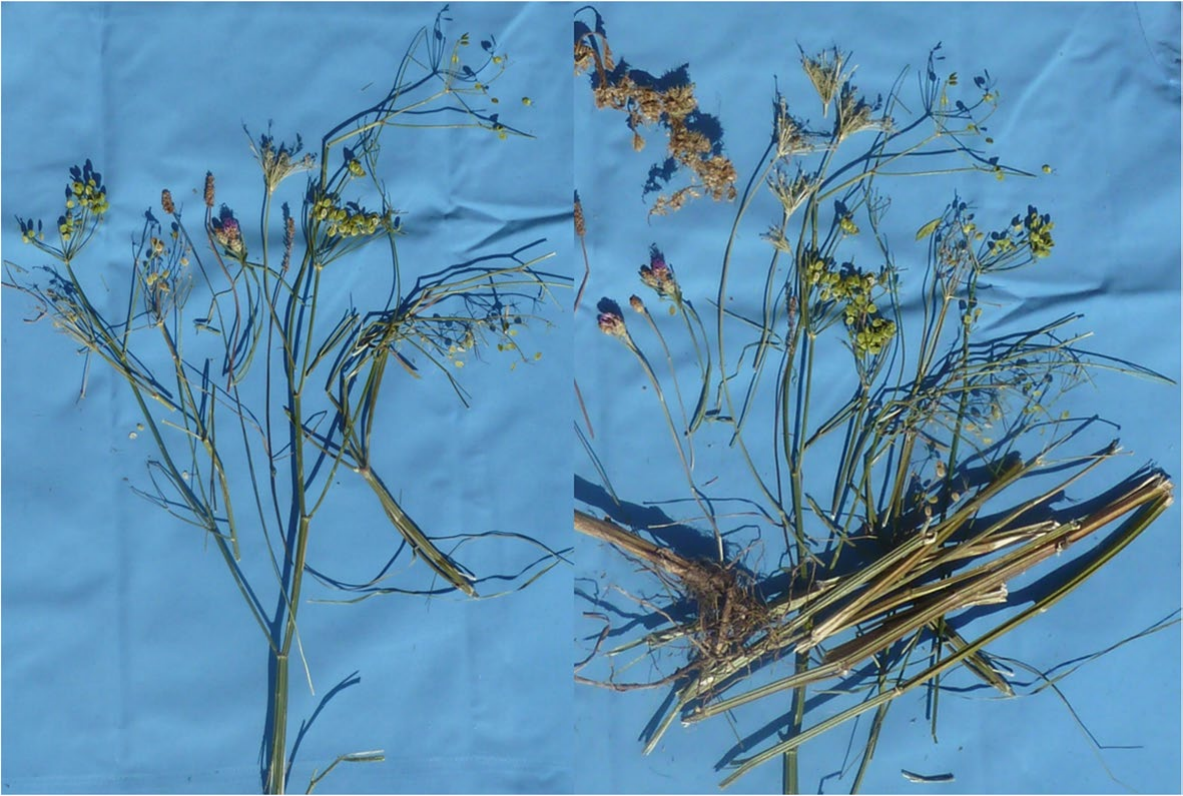
Parsnip (Pastinaca sativa) found in a hay sample from one of the stables

A thoroughbred and a German sportshorse (horse 2 and 3), both geldings, 11 and 10 years old, were presented at the clinic in April 2019, with sudden onset of depression, severe photophobia, dermatitis and corneal edema. The symptoms started in both horses about one week after starting a new hay bale, which was not fed to the other horses in this stable. The hay was purchased from the same distributor as in sTable 1. At admission, both horses had increased heart and respiratory rates with moderate depression and inappetence. The unpigmented skin at the head and prepuce showed severe crusting and oozing while the pigmented areas did not show alterations (Fig. 5). Both horses had severe blepharospasm, moderate epiphora and severe photophobia. They had a moderate bilateral conjunctivitis, uveitis and generalized corneal edema. Horse 2 had central, fluorescein positive corneal lesions (Fig. 6) while horse 3 showed a unilateral hemorrhage into the anterior chamber (Fig. 7). GGT, GLDH, AP, direct and indirect Bilirubin and bile acids were analyzed in an external laboratory^b^ and were within the reference ranges in both horses. White blood cell count, total protein and packed cell volume were measured in house^a^ and were within the reference ranges in both horses. Selenium and zinc were not measured. Botanical analysis of the hay revealed large contents of *Pastinaca sativa* in the samples. Besides long stems, high amounts of fruits (schizocarps) and a root were identified (Fig. 8). Both patients were treated symptomatically with flunixine meglumine^6^ (1.1 mg/kg BW 1–2 x/day, orally) and omeprazole^7^ (2 mg/kg BW 1x/day, orally), they were stabled and protected from sunlight. Topically, horse 2 received gentamicin^2^ (3x/day), dexpanthenol with retinolpalmitate^8^ (3x/day) and atropine^9^ (1x/day) while horse 3 received gentamicin with dexamethasone^10^ (3x/day), dexpanthenol with retinolpalmitate^8^ (3x/day), and atropine^9^ (2x/day). Skin care was provided with dexpathenol^12^. In both horses, ocular changes and dermatitis worsened for two days after admission and then improved significantly throughout the next days. They were discharged after 13 days of treatment. At that time point, horse 2 showed a mild corneal edema in the ventro-temporal areas of both eyes and the following treatment recommendations were made: topical treatment with gentamicin^2^ (3x/day for 3 days), dexpanthenol with retinolpalmitate^8^ (2x/day for one week) and a NaCl 5% solution^11^ (3x/day for one week). Horse 3 did not require further treatment as the cornea did not show any signs of edema and the unilateral hemorrhage into the anterior chamber had resolved. Horses were kept indoors for ten additional days and protected with sunscreen for one month.

### Stable 3

In April 2019, our clinic was contacted to examine six horses in a third stable in the surroundings of Berlin, which suffered from dermatitis and corneal edema. All horses were fed hay from the same distributor as horses 1–3. Symptoms appeared 3 days after starting a new hay bale. Horses were of different breeds (Hanoverian (*n* = 1); German Sportshorse (*n* = 1), Brandenburg Horse (*n* = 1); Welsh A Pony (*n* = 1), Pony (*n* = 1); Mecklenburg Horse (*n* = 1)), two were mares and four were geldings. The horses were 6–30 (median 14 ± 8.8) years old. Botanical analysis of a representative hay sample revealed similar findings as described above. Blood leukocytes, GGT and AST (measured in house^a, c^) were within the reference ranges in all horses. Other blood parameters were not measured. In relation to horses 1–3, symptoms were mild. Two Horses showed mildly enlarged mandibular lymph nodes, 4/6 horses had mild-moderate blepharospasm, 1/6 had epiphora and 1/6 showed photophobia at the time of examination. Ocular changes were blepharitis (4/6), conjunctivitis (4/6), corneal edema (1/6) and miosis or mid-sized pupils despite pretreatment with atropine (5/6). Additional signs of uveitis (e.g., cloudiness of aqueous humor/flare, reduced intraocular pressure) were not observed and topically applied corneal fluorescein retention was negative in all horses. Different degrees of skin alterations (mild-moderate hyperkeratosis, scaling and crusting) were observed in unpigmented areas of the head. Systemic treatment with flunixine meglumine (1.1 mg/kg BW 1y/day orally) was necessary in one horse. 5/6 horses received topical ocular treatment with gentamicin (3x/day), 3/6 with dexamethasone (2-3x/day) and 5/6 were treated with atropine (1-2x/day). Skin care was provided with dexpanthenol and sunscreen. Medication was provided by the referring veterinarian. Treatment duration was 9 ± 1.5 days and all horses showed complete resolution of skin and ocular lesions.

## Discussion and conclusions

In this case series, nine horses from three different stables developed photodermatitis and ocular changes after the ingestion of hay containing high amounts of wild parsnip (*Pastinaca sativa*). Reports about photosensitization after ingestion of parsnip in horses or other livestock are rare [10, 11]. Nevertheless, photodermatitis and corneal edema after ingestion of furocoumarins have been reported in sheep [12]. When exposed to UV radiation from sunlight, furocoumarins interact with oxygen and produce ROS (reactive oxygen species). These ROS can damage cell membranes in the skin and ocular tissues. Lesions are suspected to occur due to systemic uptake and direct topical contact. The two furocoumarins 5-MOP (= psoralen or bergapten) and 8-MOP are commonly used in PUVA-therapy (psolaren plus UV-A radiation, also called photochemotherapy). This therapy is used to treat skin diseases like vitiligo or psoriasis in humans and has been studied in animal models [13–16]. Barker et al. (1986) reported corneal opacities and, less frequently, lenticular opacities in mice after administration of 5-MOP and 8-MOP. Other studies described chemosis, corneal stromal edema, multiple punctate opacities in the superficial layer of the anterior cortex of the lens, cataract formation, devascularization of the iris, pupil dilation (both in combination with mature cataract), clouding of the anterior chamber and conjunctival hyperemia in the furocoumarin treated animals, while control animals did not show ocular injury [14, 15]. In the eye, the actions of furocoumarins were limited to the corneal stroma, anterior chamber, iris and anterior lens, which are the anatomic sides of long ultraviolet absorption. The maximum efficiency was found between 320–340 nm with decreasing efficiency to 330 nm and no effect for wavelengths longer than 380 nm was observed. These effects should be differentiated from those of UV-B radiation (280–315 nm wavelength). At this wavelength, very little light penetrates beyond the cornea and injuries are mostly limited to the corneal epithelium as seen in photokeratitis or “snow blindness” [16, 17]. It was stated that guinea pigs were more susceptible to photosensitizing injury with psoralen than rabbits and mice and experimental data suggest that this might be due to a difference in penetration of 8-MOP into the eye. The threshold dose of 8-MOP required to produce ocular injury in guinea pigs was in the range of 25 mg/kg body weight while in rabbits a dose of 200 mg/kg body weight was required [16]. Unfortunately, furocoumarin concentrations were not measured in the present case series due to lack of analytic equipment and invasive nature of sample collection, so it is not possible to compare equine susceptibility to the one in rodents.

In 2018, Quinn et al. developed a grading system for photodermatitis in sheep ranging from Score 0 (no apparent lesions) to 5 (severe lesions) and graded the areas “face and muzzle”, “eyes”, “ears” and “fleece/body”. Scores for the different body areas were added and a total score of < 7 rated as mild, < 12 rated as moderate and ≥ 12 as severe [18]. Unlike in sheep, ears and body were not involved in our cases. This could be due to the fact, that none of the horses had unpigmented ears or other exposed areas of the body. Unpigmented extremities were not involved, probably because they were less exposed to sunlight and did not have direct contact to parsnip. According to the grading system proposed by Quinn et al. the lesions in the areas “face and muzzle” and “eyes” would be categorized as mild in 3/9 horses, moderate in 2/9 horses and severe in 4/9 horses. They would add up to mildly affected animals in 6/9 cases and moderately affected animals in 3/9 cases. We suggest including the presence of photophobia and miosis in the description of the eye lesions (both as mild lesions) an to add an evaluation of the general condition of the animal (not affected or with signs of mild, moderate or severe depression).

In patient 1, a correct diagnosis could only be stated at the second clinic stay. Possible reasons for the delayed diagnosis might be the large areas of unpigmented skin at the head, which resulted in dermatitis involving almost the complete head. Thus, symptoms appeared similar to Pemphigus foliaceus. In horses, acantholysis can occur in Pemphigus foliaceus and secondary, after bacterial infection due to isolation of acanthocytes by bacterial proteases. Both diseases additionally share an unspecific, superficial lymphocytic dermatitis and can easily be confused in the histopathologic examination. Additionally, the analyzed hay sample did not include any plants known to cause photosensitization. This does not exclude the possibility that such plants were present in the hay but may contribute to initial diagnostic difficulties.

The diagnosis of photosensitization in horses 2–9 was made based on the history and clinical picture and was not confirmed pathologically, which is a limitation of the study. Horses 2 and 3 had extensive lesions, yet a relationship to the hay distributor from horse 1 was revealed very quickly. Together with the clinical picture the diagnosis was clear, and we did not see a benefit in a skin biopsy. The skin lesions in horses 4–9 were minor so there was no indication to biopsy.

Dermatitis caused by *Streptococcus dysgalactiae* subsp. *equisimilis*, the bacterium cultured from the skin in horse 1, has been described in children and, in rare cases, in adults [18–21]. The bacterium colonizes many mucosal surfaces of humans and was previously considered nonpathogenic. However, many recent studies revealed that *Streptococcus dysgalactiae* subsp. *equisimilis* can cause various soft-tissue infections and occasionally more serious infections including neonatal sepsis, bacteremia, endocarditis, meningitis, peritonitis and arthritis [21, 22]. In horses, *Streptococcus dysgalactiae* subsp. *equisimilis* infections have been considered as very infrequent and opportunistic with the highest isolation rate from the reproductive tract. Sampling with nasopharyngeal swabs in healthy horses revealed growth of *Streptococcus dysgalactiae* subsp. *equisimilis* in 23.7% of the cases [19]. Differences in pathogenicity of the bacterium in humans and horses could be due to discrepancies in streptokinase that shows only a 25.4% identity between both species [20]. Additionally, the type of *Streptococcus dysgalactiae* subsp. *equisimilis* and the amount of colony forming units might play a role in expression of clinical symptoms. It is unlikely that *Streptococcus equi* subsp. *equisimilis* played a relevant role in the present case.

Wild parsnip is widespread in Europe and the hay distributor reported that it was growing in the field where the relevant hay was harvested, but usually in very low amounts. In the year of 2018, the relation of grasses and wild parsnip changed in favor of parsnip. In Germany and other European countries, the years 2017/2018 were particularly difficult for hay production due to heavy rain in the summer of 2017 and severe draught in 2018. As a result, hay quality was low, prices increased, and hay producers were pressured to use all available grassland for hay production. Hence, weather resistant plants may have gained advantage in growth, leading to higher concentrations of uncharacteristic plants in hay and silage. Horse owners, caretakers and veterinarians are usually confronted with plants from hay samples in their dried condition, without any access to fresh plant material for comparison. This complicates correct identification and may lead to confusions. In dried condition, parsnip might be confused with other plants of the Umbelliferae family such as common hogweed (*Heracleum sphodylium),* giant hogweed (*Heracleum mantegazzianum*) or bishop's weed (*Ammi majus)* all known to cause photosensitization. The giant hogweed can easily be identified by its size, while the fruits of bishop's weed differ significantly from wild parsnip. Other, toxic genera such as hemlock (*Conium maculatum*), water hemlock (*Cicuta*) or fool's parsley (*Aethusa cynapium)* can also be distinguished from wild parsnip by the shape of their fruits. Hogweed fruits on the opposite look very similar to wild parsnip. When fresh, both plants can easily be distinguished by the color of their blossoms: parsnip blooms yellow while, hogweed produces white blossoms [12, 23]. As *Heracleum sphodylium* also contains furocoumarins and is known to cause photodermatitis in humans, horses and other animals, the final classification might not be necessary for adequate treatment of the patient. Animals ingesting plants containing furocoumarins frequently develop ocular alterations. This is not observed in animals showing symptoms of photosensitivity after ingesting other types of plants such as St. John’s wort. Thus, ocular changes combined with symptoms of photosensitivity may be the cardinal symptom for intoxications with furocoumarins.

## Data Availability

All data generated or analyzed during this study will be available from the corresponding author on reasonable request.

## Abbreviations

ACTH: Adrenocorticotropic hormone
AST: Aspartate-aminotransferase
BW: Body weight
GGT: Gamma-glutamyltransferase
GLDH: Glutamatedehydrogenase
subsp: Subspecies

## Declarations

### Manufacturer's addresses

^a^VetScan HM 5, Scil animal care company GmbH, Viernheim, Germany

^b^Laboklin GmbH & Co. KG, Bad Kissingen, Germany; certified according to DIN EN ISO/IEC 17025:2005

^c^Reflotron® plus Roche Deutschland Holding GmbH, Grenzach-Wyhlen/Germany

^1^Equisolon, Böhringer Ingelheim Vetmedica GmbH, Ingelheim, Germany

^2^Gent-Ophtal®, Ursapharm Arzneimittel GmbH, Saarbrücken, Germany

^3^ProntoVet®, B.Braun Melsungen AG, Melsungen, Germany

^4^Navalis Orthosal® Selen, Navalis® nutraceuticals GmbH, Filderstadt

^5^Azathioprin HEXAL® 100 mg, Hexal AG, Holzkirchen, Germany

^6^Flunidol® 5%, cp-pharma Handelsgesellschaft mbH, Burgdorf, Germany

^7^Equizol®, cp-pharma Handelsgesellschaft mbH, Burgdorf, Germany

^8^Vitamycin® Augensalbe, cp-pharma Handelsgesellschaft mbH, Burgdorf, Germany

^9^Atropin-POS 0,5 %, Ursapharm Arzneimittel GmbH, Saarbrücken, Germany

^10^Dexa-Gentamicin, Ursapharm Arzneimittel GmbH, Saarbrücken, Germany

^11^Omni-Sorb®, Omnivision GmbH, Wien, Austria

^12^Panthenol-ratiopharm® Wundbalsam, ratiopharm GmbH, Ulm, Germany
